# Molecular Mechanisms of Exercise on Cancer: A Bibliometrics Study and Visualization Analysis *via* CiteSpace

**DOI:** 10.3389/fmolb.2021.797902

**Published:** 2022-01-13

**Authors:** Dongling Zhong, Yuxi Li, Yijie Huang, Xiaojuan Hong, Juan Li, Rongjiang Jin

**Affiliations:** ^1^ School of Health Preservation and Rehabilitation, Chengdu University of Traditional Chinese Medicine, Chengdu, China; ^2^ School of Acupuncture Moxibustion and Tuina, The Third Affiliated Hospital, Chengdu University of Traditional Chinese Medicine, Chengdu, China; ^3^ The Seventh Affiliated Hospital, Sun Yat-sen University, Shenzhen, China

**Keywords:** exercise, cancer, molecular mechanisms, bibliometrics, visualization analysis, citespace

## Abstract

**Objective:** To analyze the research hot spots and frontiers of molecular mechanisms of exercise on cancer *via* CiteSpace.

**Method:** Related publications in the Web of Science Core Collection Science Citation Index Expanded were retrieved from inception to November 27th, 2021. Then we used CiteSpace to generate network maps and identify top authors, institutions, countries, keywords, co-cited authors, journals, references and research trends.

**Results:** A total of 1,130 related publications were retrieved. The most productive author and journal were Lee W Jones and PLOS ONE. Hanahan D and Warburg O were the most cited authors. Fudan University and Shanghai Jiao Tong University were the leading institutions, while China was the leading country. Top-cited authors and references generally focused on the epidemiology and hallmarks of cancer. Top five keywords with both high frequency and high betweenness centrality were breast cancer, aerobic glycolysis, oxidative stress, gene expression, skeletal muscle. Keyword “warburg effect” ranked first with the highest citation burst, while “inflammation”, “hepatocellular carcinoma”, “epithelial mesenchymal transition”, and “adipose tissue” were emerging research foci.

**Conclusion:** This study analyzed the research hot spots and frontiers of molecular mechanisms of exercise on cancer *via* CiteSpace. Based on the results, altered metabolism (aerobic glycolysis, insulin resistance, myokines), oxidative stress, gene expression and apoptosis were hot-research mechanisms of exercise on cancer. Emerging research foci of mechanisms were generally around inflammation, epithelial mesenchymal transition and adipokines. In addition, future studies could carry in-depth research of interactions between different mechanisms and try to elucidate the recommended doses and intensities of exercise for cancer, especially in breast, colorectal, prostate cancer and hepatocellular carcinoma.

## Introduction

Cancer is one of the leading causes of disability and mortality worldwide. According to latest estimates of the International Agency for Research on Cancer (IARC) (https://www.iarc.who.int/), there were about 19.3 million new cases of cancer, 10 million cancer deaths worldwide in 2020. In 2040, there will be 28.4 million new cancer cases.

Exercise plays an essential role in the management of cancer, especially in cancer prevention, cancer progression control, cancer-related outcomes improvement (Galvao et al., 2010; Speck et al., 2010; Magne et al., 2011). According to a study involving 430,000 people, leisure-time physical activity was associated with lower risks of several cancer types (Moore et al., 2016). In addition, exercise could reduce cancer risk factors such as obesity (Swift et al., 2018), inflammation (Metsios et al., 2020) and improve health-related outcomes in cancer survivors (Campbell et al., 2019). On the contrary, physical inactivity could increase the risk of colon cancer (Wolin et al., 2009) and breast cancer (Kyu et al., 2016). Therefore, multiple organizations including the American Cancer Society (ACS) (Rock et al., 2012), the American College of Sports Medicine (ACSM) (Schmitz et al., 2010), Exercise and Sports Science Australia (ESSA) (Hayes et al., 2019) have published exercise guidelines for cancer survivors.

Due to the rapid development of technologies, the possible molecular mechanisms of exercise are being illuminated, which may be possibly related to changes in the serum markers level, inflammation markers, oxidative stress and so on (Hojman et al., 2018). However, research hot spots and frontiers of this field remain unclear.

CiteSpace is a Java-based application to analyze and visualize the hot spots and research frontiers in the scientific literature of a discipline or knowledge domain in a certain period with metrology, co-occurrence analysis and cluster analysis (Chen, 2004; Chen, 2006). In this study, we intended to analyze the hot spots and research frontiers of molecular mechanisms of exercise on cancer via CiteSpace, which may help us understand the curative and preventive effects of exercise for cancer better.

## Methods

### Search Strategy

We retrieved articles in the Web of Science Core Collection Science Citation Index Expanded (SCI-Expanded) from inception to November 27th, 2021, using the following terms: exercise, neoplasms and molecular mechanism. Table 1 shows the detailed search strategy.

**TABLE 1:** Search strategy.

Set	Search query
#1	TS=(cancer* or tumor* or tumour* or neoplas* or malignan* or carcinoma* or adenocarcinoma* or choricarcinoma* or leukemia* or leukaemia* or metastat* or sarcoma* or teratoma* or melanoma* or lymphoma* or myeloma*)
#2	{TS=[physical* near/5 (fit* or activit* or movement*)]} OR [TS=(exercis* or aerobic* or walk* or endurance* or training or tai ji or yoga or tai-chi or tai-ji or tai chi or taiji* or pilates)]
#3	(#2) AND (#1)
#4	[TS=(molecular mechanism*)] AND (#3)

### Inclusion and Exclusion Criteria

Peer-reviewed published original articles or reviews about the molecular mechanisms of exercise on cancer were included.

Exclusion criteria were: 1) conference abstracts or corrigendum documents; 2) unpublished articles; 3) repeated publications; 4) unrelated articles.

### Bibliometrics and Visualization Analysis

We exported retrieved articles in plain text format with full records and references, named “download_XXX.txt” and then imported into CiteSpace 5.8.R3 for further analysis. When mapping visualization knowledge figures, we followed the main procedural steps of CiteSpace, including time slicing, thresholding, modeling, pruning, merging, and mapping (Chen, 2004). Central concepts of CiteSpace includes burst detection, betweenness centrality, and heterogeneous networks, which can help to timely visualize the research status, hot spots, and frontiers (Chen, 2006). Nodes in different maps represent authors, institutions, countries or keywords. Size of nodes indicates the frequency of occurrence or citation, and color of nodes indicated the occurrence or citation years. Besides, nodes with purple trims suggests high betweenness centrality, which are often identified as hot spots or turning points in a field.

## Results

### Distribution of Articles by Publication Years

**FIGURE 1:**
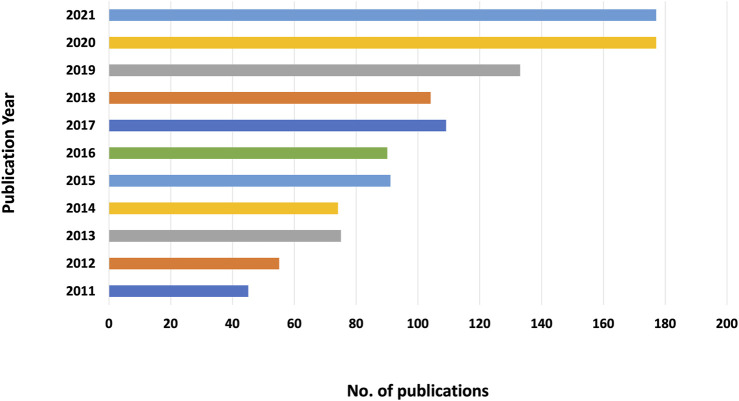
Annual trend of publications.

After removing 61 unqualified records, 1,130 articles were obtained. Figure 1 shows that the number of publications has generally increased with some fluctuations, ranging from 45 to 177 publications. From 2019 to 2020, the number of related publications increased the most (33 publications), indicating that more and more researchers are beginning to pay attention to this field.

### Co-Authorship, Co-Institution, and Co-Country

We analyzed publications with time slicing of 1 year and the top 50 levels of most-cited or occurred items from each slice. Tree ring history was selected as node display pattern. Lines between nodes represent cooperation, and the color of lines indicates the first cooperation year.

**FIGURE 2:**
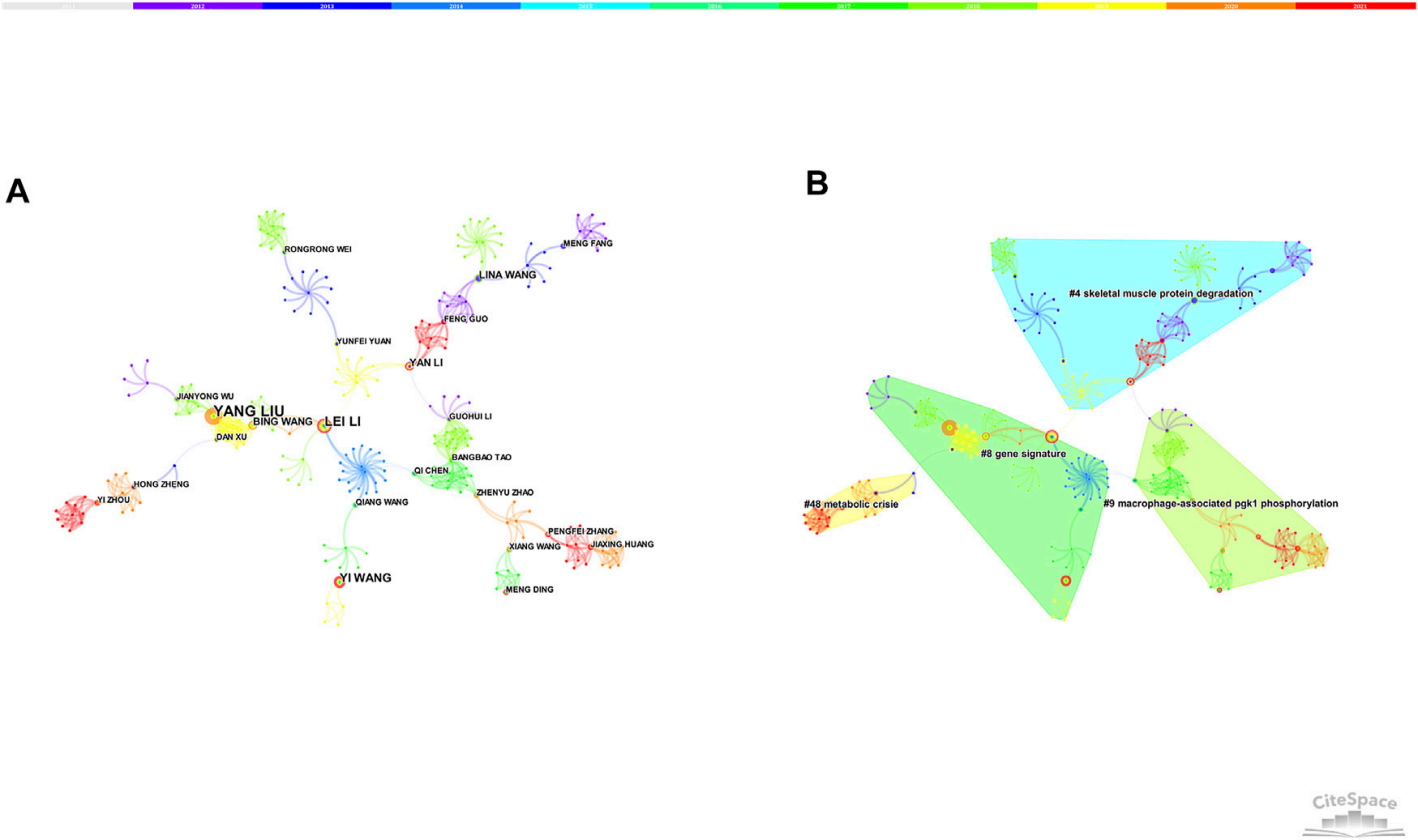
**(A)** The network of co-authorship. **(B)** The network of co-authorship clusters.

7,588 nodes and 23,925 links composed of the merged co-authorship network, and we chose to visualize the largest connected component only (Figure 2A). The co-authorship network shows prolific authors and the collaboration among them. The most productive author was Lee W Jones with a total of 8 articles, followed by Jing Li (6 articles) and Yang Liu (6 articles). Although many authors have published relevant articles, there was little collaboration among them. Besides, the centrality of authors was relatively low, suggesting that more high-quality and large-scale collaborations are needed in the future.

In general, the silhouette value is used to evaluate the clusters. If the silhouette value is over 0.7, the cluster is efficient and convincing. Four clusters (the silhouette value of 4 clusters exceeded over 0.9) were produced by log-likelihood ratio, mainly around skeletal muscle protein degradation, gene signature, metabolic crisis, macrophage-associated PGK-1 phosphorylation (Figure 2B).

**FIGURE 3:**
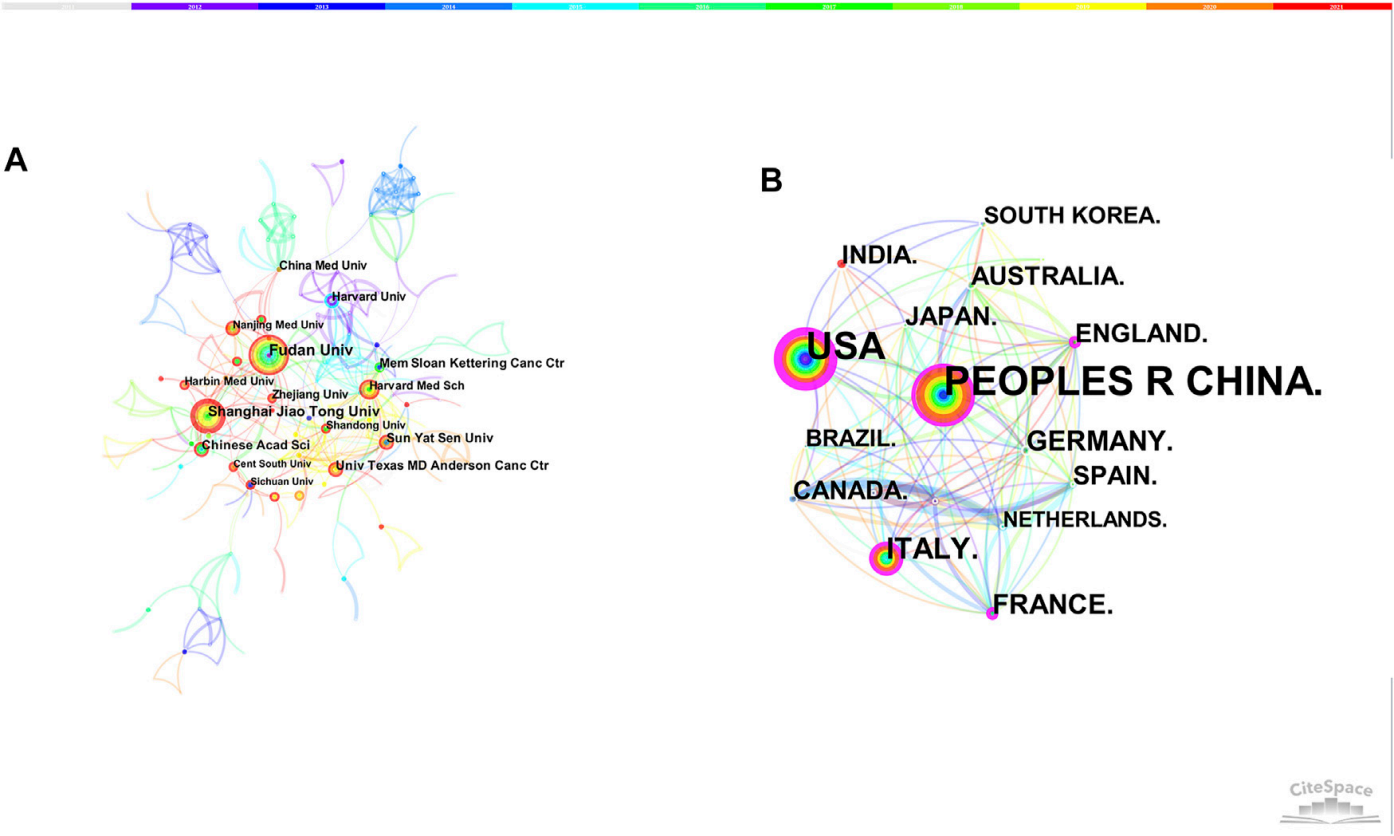
**(A)** The network of co-institution. **(B)** The network of co-country.

The merged co-institution network map is shown in Figure 3A, with 1,568 nodes and 5,227 links. The top five institutions were Fudan University (36 articles), Shanghai Jiao Tong University (36 articles), Sun Yat-sen University (22 articles), Chinese Academy of Sciences (21 articles) and Memorial Sloan Kettering Cancer Center (19 articles).

The merged co-country network consists of 86 nodes and 433 links. People’s Republic of China was the leading country (427 articles), followed by United States (316 articles), Italy (87 articles), Germany (57 articles) and France (51 articles). Except for Germany, the betweenness centrality of other countries were both over 0.1, illustrating the important contribution of these countries in this area (Figure 3B).

### Author and Journal Co-Citation

**FIGURE 4:**
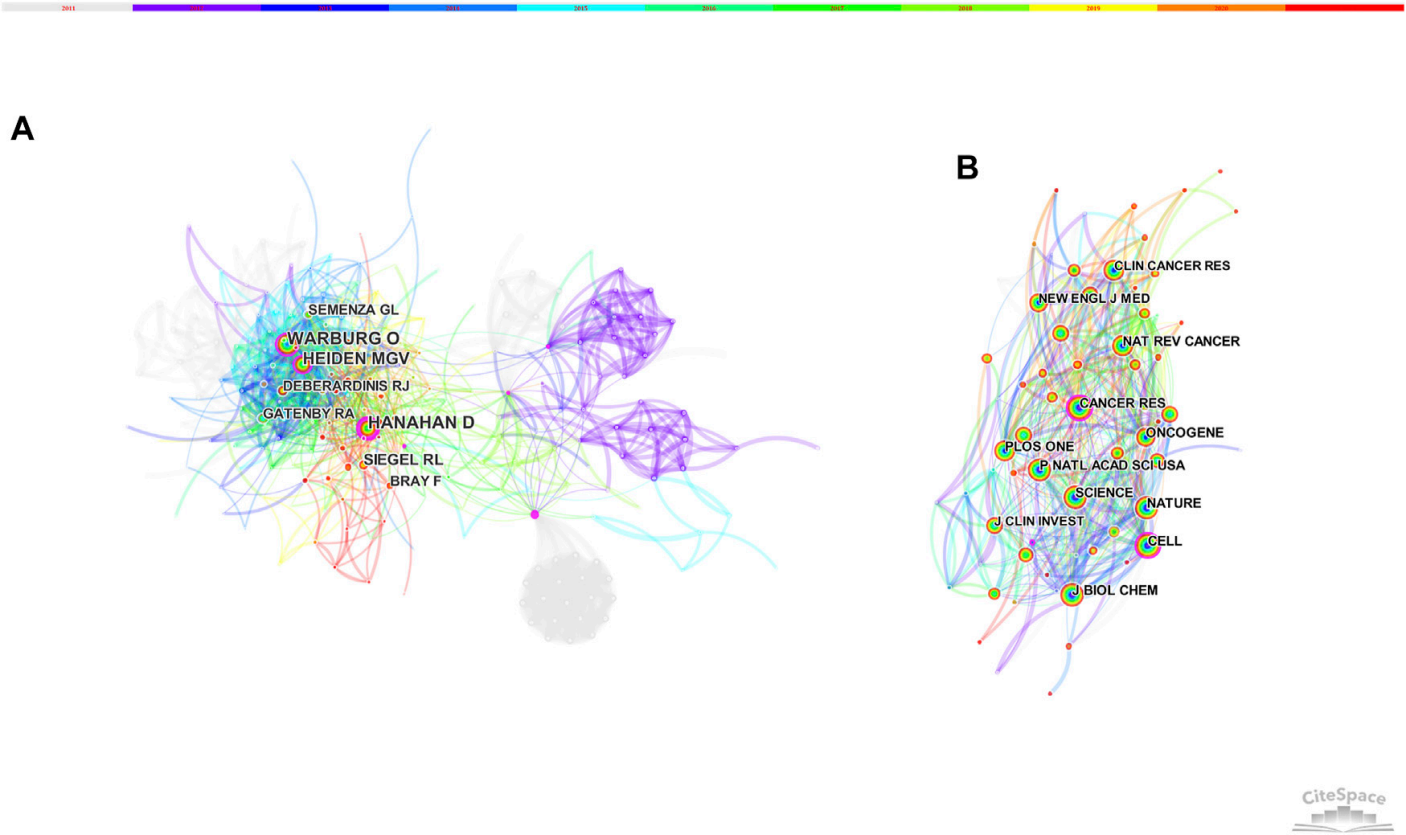
**(A)** The network of author co-citation. **(B)** The network of journal co-citation.

Figure 4 displays the network of author and journal co-citation. The most-cited authors were Hanahan D (130 citations) and Warburg O (130 citations), followed by Heiden MGV (119 citations), Siegel RL (74 citations), Semenza GL (62 citations) and Deberardinis RJ (61 citations). Besides, Hanahan D, Warburg O and Heiden MGV were also high-centrality authors (Figure 4A).

The top-ranked journal by citation counts was PLOS ONE with 638 citations, followed by Nature (634 citations), Cancer Research (629 citations), Proceedings of the National Academy of Sciences of the United States of America (627 citations) and Cell (587 citations) (Figure 4B). Besides, Cancer Research and Cell got both high frequency and high betweenness centrality, indicating its critical role in this field (Table 2).

**TABLE 2:** The top five cited journal.

Rank	Frequency	Cited journal	IF	Centrality	Cited journal	IF
1	638	PLOS ONE	3.2	0.15	Cancer research	12.701
2	634	Nature	49.962	0.14	Cell	41.584
3	629	Cancer research	12.701	0.1	The biochemical journal	3.857
4	627	Proceedings of the national academy of sciences of the United States of America	9.58	0.09	The journal of clinical investigation	14.808
5	587	Cell	41.584	0.08	International journal of cancer	7.396

### References Co-Citation

**FIGURE 5:**
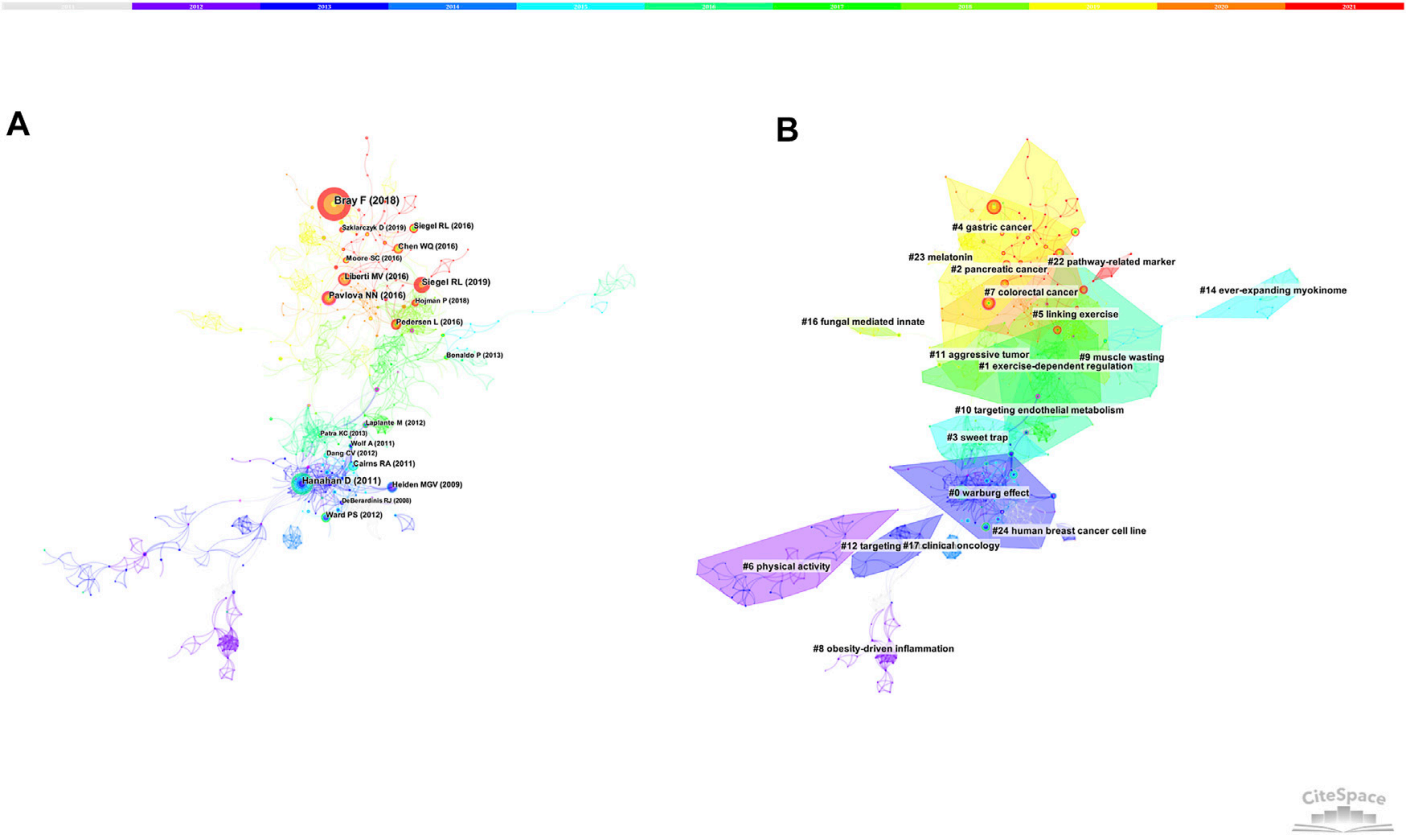
**(A)** The network of co-cited references. **(B)** The network of co-cited references clusters.

Figure 5A and Table 3 show the top co-cited references with high frequency and high betweenness centrality. The first co-cited reference was published by Bray F et al., which provided a status report on the global burden of cancer (Bray et al., 2018), The reference published by Hanahan D introduced the hallmarks of cancer, which may affect the development of anti-cancer therapies (Hanahan and Weinberg, 2011). Siegel RL provided the cancer statistics of United States in 2019 (Siegel et al., 2019). Pavlova NN made a summary of the emerging 6 hallmarks of cancer metabolism (Pavlova and Thompson, 2016). Liberti MV summarized the explanations and controversies for the function of Warburg Effect (Liberti and Locasale, 2016).

**TABLE 3:** The top five cited references.

Rank	First author	Country	Frequency	Centrality	Year	Cited references	Journal	IF
1	Bray F	France	53	0	2018	Global cancer statistics 2018: GLOBOCAN estimates of incidence and mortality worldwide for 36 cancers in 185 countries	CA: a cancer journal for clinicians	508.702
2	Hanahan D	Switzerland	35	0.06	2011	Hallmarks of cancer: the next generation	Cell	41.584
3	Siegel RL	United States	26	0.01	2019	Cancer statistics, 2019	CA: a cancer journal for clinicians	508.702
4	Pavlova NN	United States	24	0.11	2016	The emerging hallmarks of cancer metabolism	Cell Metabolism	27.287
5	Liberti MV	United States	21	0.02	2016	The warburg effect: How does it benefit cancer cells?	Trends in biochemical sciences	13.807

Other high-cited publications were as follows: Pedersen L et al. revealed that exercise-induced muscle-derived interleukin-6 (IL-6) was involved in natural killer (NK) cell redistribution, thus reduced the incidence and growth of cancer (Pedersen et al., 2016). Hojman P et al. concluded that the tumor growth-inhibitory effect of exercise was probably mediated by several different mechanisms (the cellular immune system and exercise-induced myokines) (Hojman et al., 2018). Moore SC et al. found that leisure-time physical activity was associated with lower risks of many cancers (Moore et al., 2016).

**FIGURE 6:**
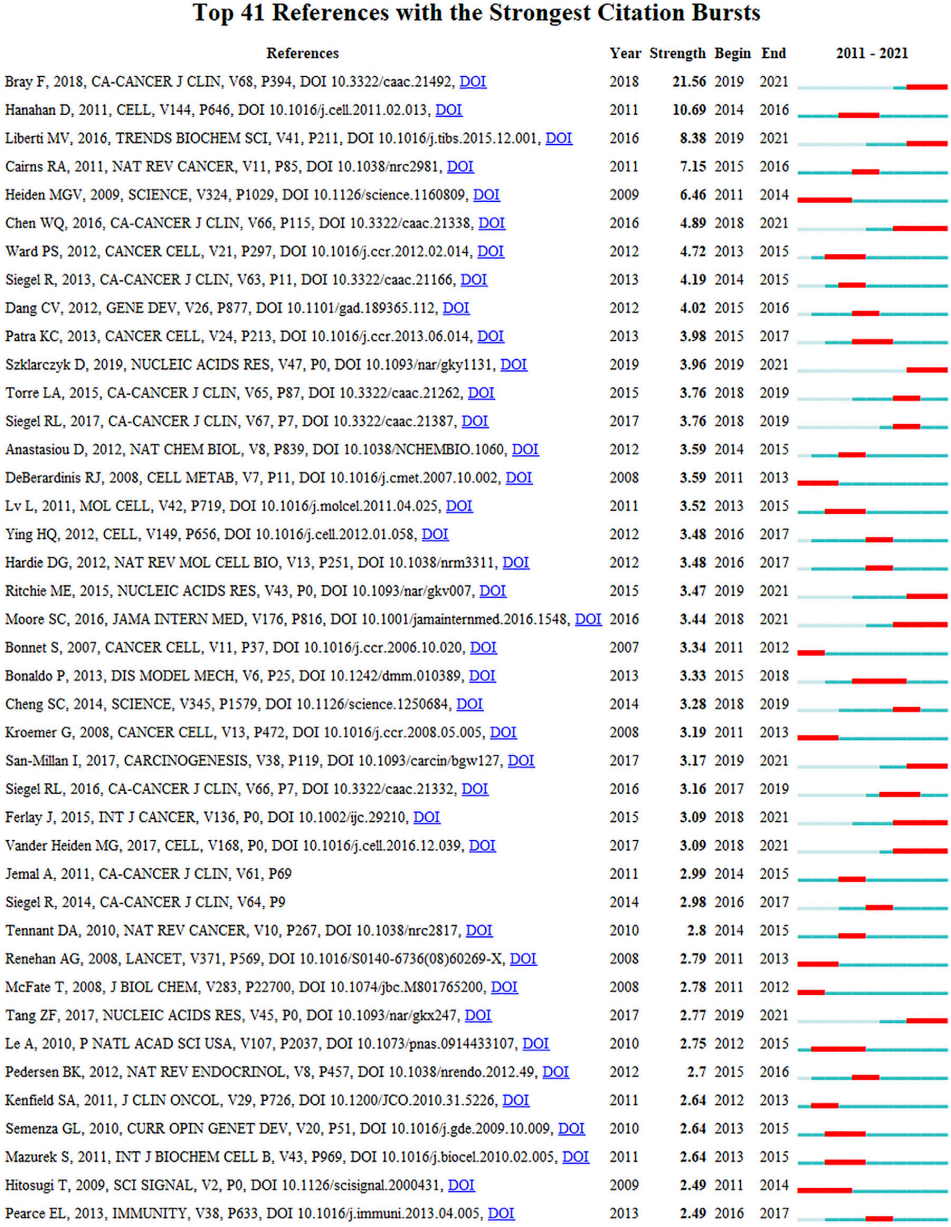
Top 41 references with strongest citation bursts.

Figure 5B shows the largest 19 clusters of co-cited references. Figure 6 displays the top 41 references with strongest citation bursts, which indicating the emerging trends or increasing interests in the field. Generally, the most co-cited references usually got the strongest citation bursts.

### Keywords Co-Citation and Clusters

**FIGURE 7:**
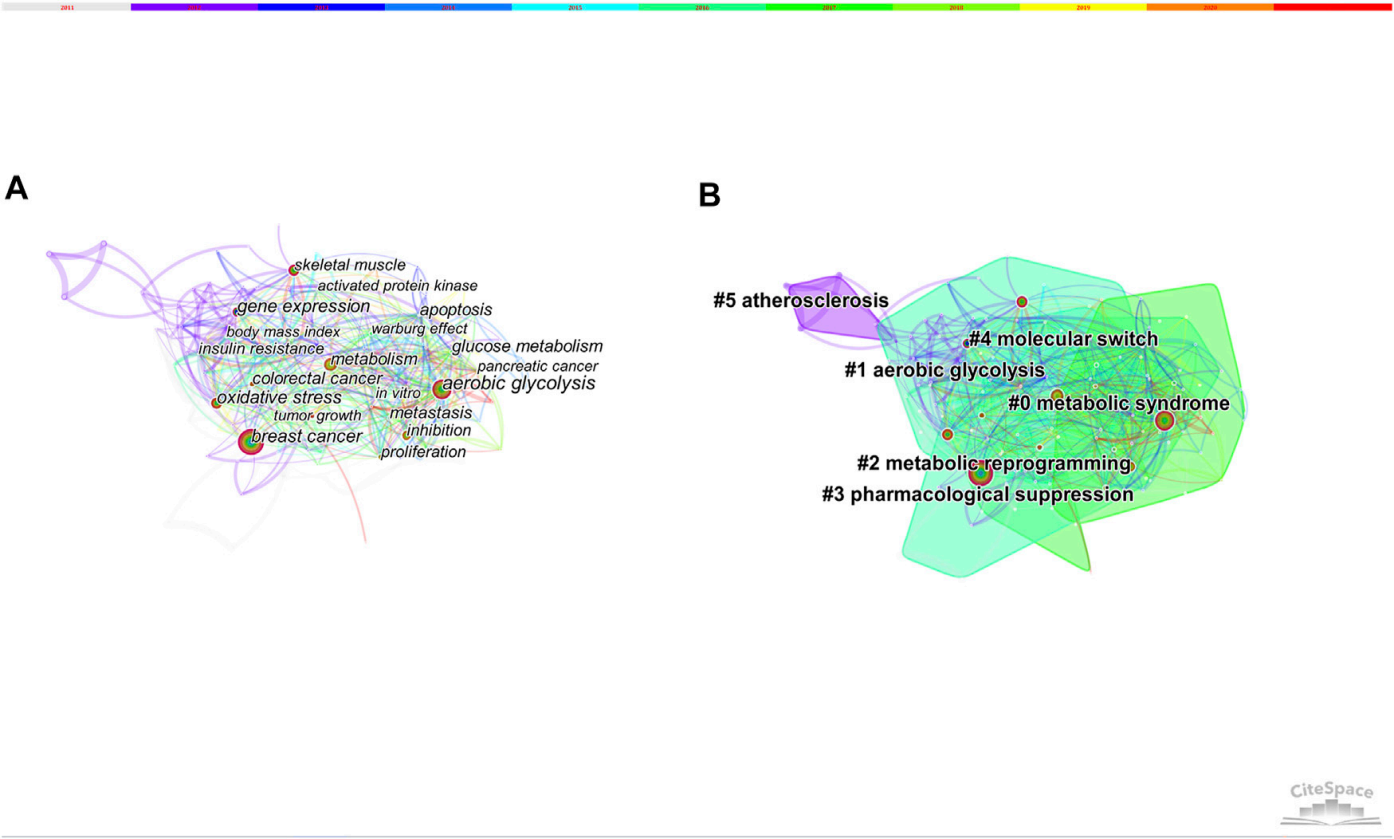
**(A)** The network of co-occurring keywords. **(B)** The network of co-occurring keywords clusters.

Keywords are the high-level summary. High-frequency and high-centrality keywords often reflect hot research topics in this field. We analyzed publications with time slicing of 1 year and the top 30 levels of most-cited or occurred items from each slice. 149 nodes and 1,138 links composed of the merged co-occurring keywords network. Table 4 presents the top 10 keywords with high frequency and centrality. 6 clusters were produced by log-likelihood ratio, including metabolic syndrome, aerobic glycolysis, metabolic reprogramming, pharmacological suppression, molecular switch and atherosclerosis (Figure 7).

**TABLE 4:** Top 10 keywords in terms of frequency and centrality.

Rank	Frequency	Keywords	Centrality	Keywords
1	128	Breast cancer	0.18	Oxidative stress
2	113	Aerobic glycolysis	0.14	Breast cancer
3	83	Oxidative stress	0.14	Aerobic glycolysis
4	80	Gene expression	0.14	Gene expression
5	69	Skeletal muscle	0.12	Skeletal muscle
6	69	Metabolism	0.07	Colorectal cancer
7	45	Colorectal cancer	0.06	Apoptosis
8	40	Apoptosis	0.06	Prostate cancer
9	32	Hepatocellular carcinoma	0.05	Insulin resistance
10	30	Insulin resistance	0.04	Metabolism

### Keywords With Citation Bursts

**FIGURE 8:**
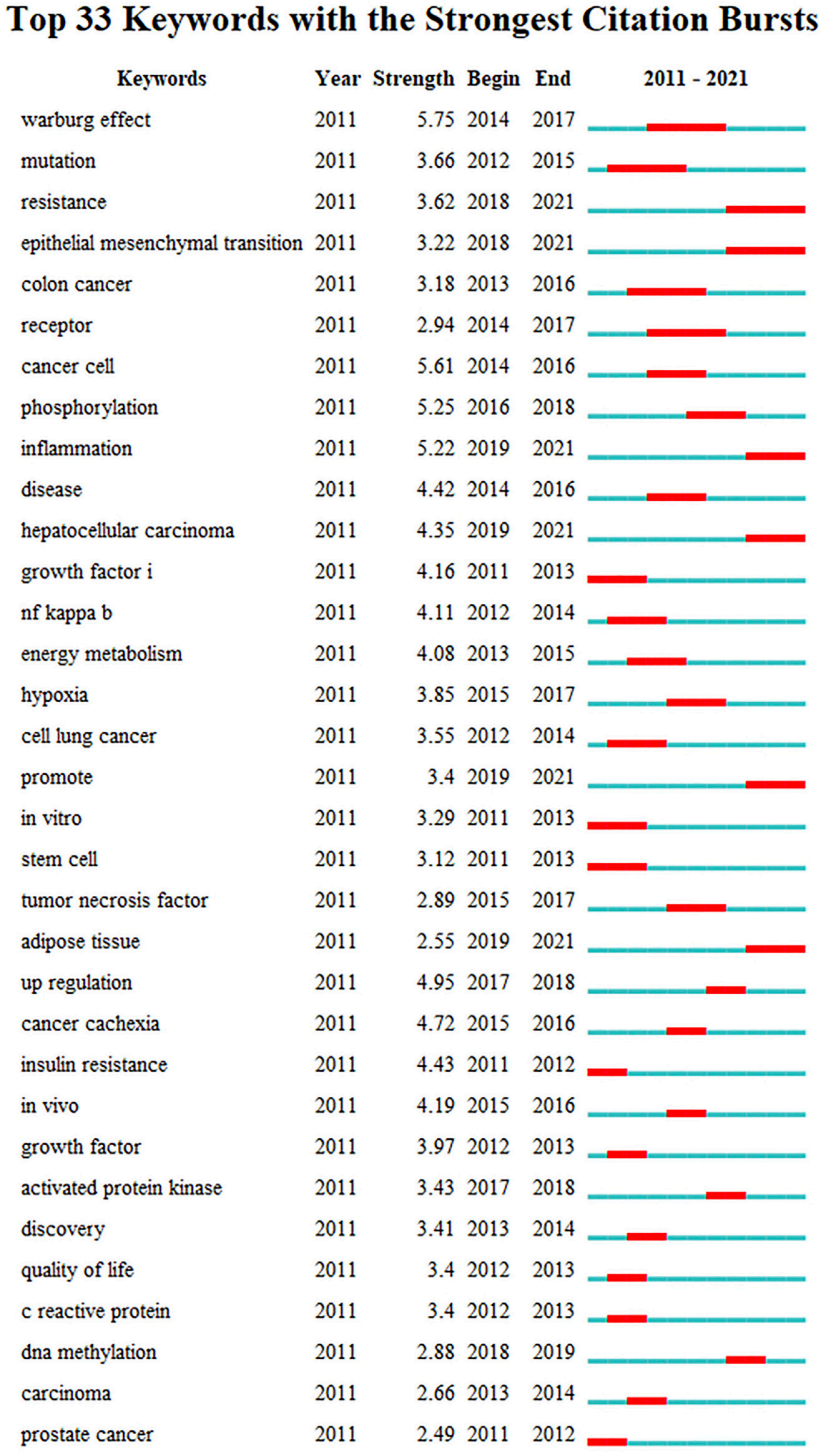
Top 33 keywords with strongest citation bursts.

Figure 8 presents the top 33 keywords with citation bursts. The blue line indicates the time interval, while the red line indicates the time period when a keyword had a burst (Chen et al., 2014). Keywords “warburg effect” with the strongest citation bursts appeared in 2014, indicating the importance of glucose metabolism in cancer cells. The most recent keywords with citation bursts occurred in 2019 were “inflammation”, “hepatocellular carcinoma” and “adipose tissue”. In addition, “epithelial mesenchymal transition” also continued to 2021.

## Discussion

### Summary of Findings

A total of 1,130 related articles were retrieved eventually. In the past several years, increasing researchers have begun to study the molecular mechanisms of exercise on cancer, forming a group of prolific authors represented by Lee W Jones, Jing Li and Yang Liu. China was the leading country in this field and Chinese universities have published many relevant studies. Top-cited authors and references generally focused on the epidemiology and hallmarks of cancer. Top five keywords with both high frequency and high betweenness centrality were breast cancer, aerobic glycolysis, oxidative stress, gene expression, skeletal muscle. Keyword “warburg effect” ranked first with the highest citation burst, while “inflammation”, “hepatocellular carcinoma”, “epithelial mesenchymal transition”, and “adipose tissue” were emerging research foci.

### Research Hotspots on Molecular Mechanisms of Exercise on Cancer

**FIGURE 9:**
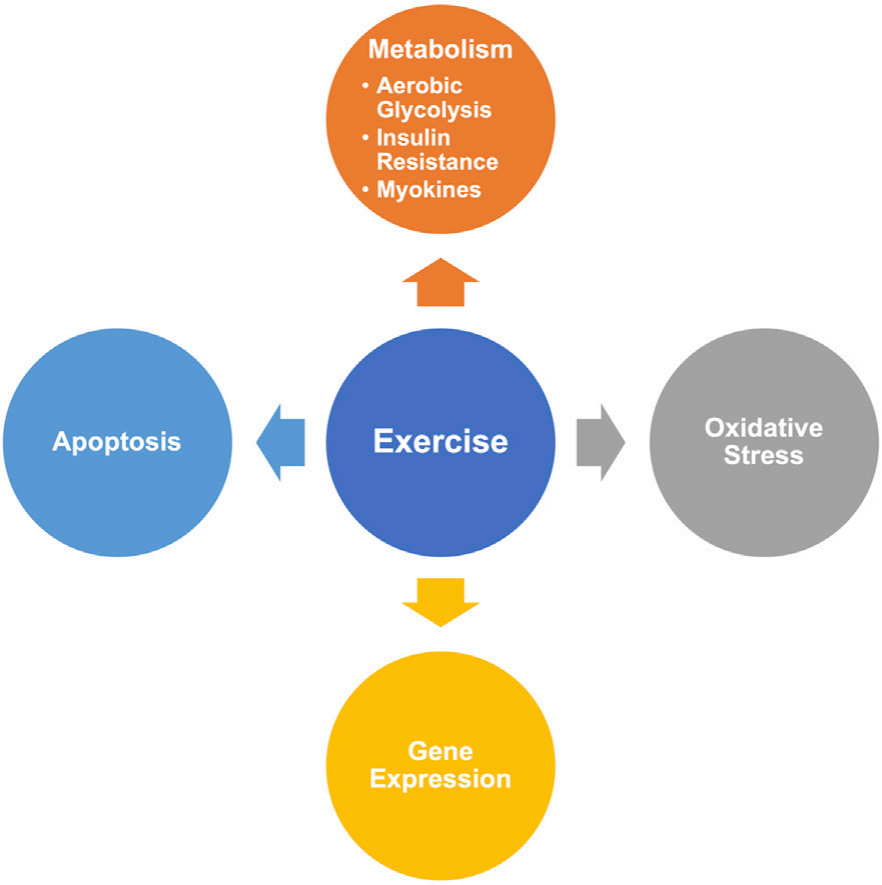
Summary of the hot-research mechanisms.

Based on the results of CiteSpace, we summarized the hot-research molecular mechanisms of exercise on cancer (Figure 9).

#### Metabolism

##### Aerobic Glycolytic/Warburg Effect

Altered glycolysis/TCA cycle has been proved to be one of hallmarks of cancer (Liberti and Locasale, 2016; Pavlova and Thompson, 2016). Highly up-regulated glycolytic phenotype could induce local acidosis, which is conducive to tumor development and invasion. Warburg effect is characterized by accelerated aerobic glycolytic metabolism even if there is sufficient oxygen supply, and is common in most malignant tumors (Cairns et al., 2011). In healthy athletes, increased systemic lactate levels during repeated high-intensity anaerobic exercise could inhibit glycolysis and net lactate production (Hollidge-Horvat et al., 1999). Evidence indicated that high-intensity anaerobic exercise was shown to inhibit the Warburg-type highly glycolytic (Hofmann, 2018). Animal studies also demonstrated that high-intensity anaerobic exercise training may have stronger effects on tumor growth compared with moderate-intensity aerobic exercise (Bacurau et al., 2007; de Lima et al., 2008; Paceli et al., 2012). However, the correlation between the host and cancer metabolism is related to the duration, time, intensity, and movement mode of exercise (Koelwyn et al., 2017).

##### Insulin/Insulin-Like Growth Factors

Insulin and the insulin-like growth factors (IGFs) family play an essential role in regulating cell growth and apoptosis, as well as proliferation and differentiation of cancer cells (Hankinson et al., 1998; Christopoulos et al., 2015). Besides, insulin-like growth factor binding protein 3 (IGFBP-3) could inhibit cell proliferation and promote cell apoptosis by regulating local IGF-1 concentration and the anti-apoptosis effect of IGFBP-3 can be inhibited in breast cancer cells (Kim et al., 2004). Irwin et al. revealed that the decrease in IGF-I and IGFBP-3 caused by exercise may explain the link between higher levels of physical activity and the survival rate of breast cancer patients (Irwin et al., 2009). Another study indicated that changes in insulin levels and/or changes in body fat or fat deposition by exercise may mediate breast cancer prognosis in part (Ligibel et al., 2008).

#### Myokine/Apoptosis

During exercise, paracrine factors coordinate tissue remodeling in response to skeletal muscle contraction. Factors secreted from muscle cells may influence cancer cell growth. Lack of physical activity probably leads to changes in myokine response. Evidence showed that myokines, such as the IL-6 superfamily, may mediate some of the inhibitory effects of exercise on mammary cancer cell proliferation (Hojman et al., 2011). Moreover, exercise can induce apoptosis of tumor cells in skeletal muscle. DNA microarrays were used to compare the transcriptome of muscle tissue in young and old mice (sedentary and exercised), and the results showed that exercise was able to stimulate the secretion of secreted protein acidic and cysteine-rich (SPARC) from muscle tissues and SPARC could inhibit colon tumorigenesis by increasing apoptosis (Aoi et al., 2013). Animal studies also suggested that moderate-intensity training may inhibit cancer cell proliferation and induce apoptosis (Westerlind et al., 2002).

#### Oxidative Stress

High levels of oxidative stress is another hallmark of cancer (Cairns et al., 2011), which is caused by the imbalance between the production and elimination of reactive oxygen species (ROS). Increased ROS levels are generally detrimental to cells, and could promote tumor formation via inducing DNA damage, pro-inflammatory cytokines (Naik and Dixit, 2011) and activating the nuclear factor-κB (NF-κB) pathway (Gloire et al., 2006). Studies showed that exercise was able to improve antioxidation and counteract the negative consequences of oxidative stress by modulating systemic oxidative status (SOS) and DNA repair capability (Tomasello et al., 2017; Simioni et al., 2018). However, controversy exists since strenuous exercise may enhance oxidative stress.

#### Cancer Types

According to the high frequency keywords, breast cancer, colorectal cancer and hepatocellular carcinoma are main research cancer types of mechanisms of exercise. Possible underlying mechanisms of breast cancer includes a reduction of sex hormones, metabolic hormones, adipokines and oxidative stress, and an improvement of the immune function (de Boer et al., 2017). Hayes et al. also outlined the mechanisms of exercise for colorectal and prostate cancer through harnessing the immune system (Hayes et al., 2016; Song and Chan, 2018). Besides, hepatocellular carcinoma was one of the most recent keywords with citation bursts, which has attracted the attention of researchers. Exercise can attenuate the progression of hepatocellular carcinoma related to changes in key signaling pathways, cellular proliferation, tumor vascularization, and necrosis (Saran et al., 2018).

### Emerging Areas on Molecular Mechanisms of Exercise on Cancer

From the citation bursts analysis, the most recent keywords with citation bursts occurred in 2019 was inflammation. Chronic inflammation is bound up with the development and progression of cancer (Gleeson et al., 2011; Friedenreich et al., 2012). Numerous molecules such as tumor necrosis factor (TNF)-α, interleukin-1 (IL-1) and IL-6 are common biomarkers of inflammation associated with cancer, and they could be regulated by the transcription factor NF-κB (Aggarwal et al., 2009). Khosravi et al. has proved the anti-inflammatory effects of exercise, especially in prostate and breast cancer survivors (Khosravi et al., 2019). Besides, recent studies suggested that obesity related-excess adipose tissue could promote the neoplasia and progression of tumor through adipose tissue inflammation (circulating cytokines such as TNF-α and IL-6) (Campbell et al., 2009; Iyengar et al., 2013). Accompanied by weight loss, exercise could attenuate adipose tissue inflammation in aged 20–40 years, overweight men (Auerbach et al., 2013; Ahmadizad et al., 2014) and obese postmenopausal breast cancer survivors (Dieli-Conwright et al., 2018).

The NF-κB family is composed of transcription factors and plays a complex and key role in the regulation of immune responses and inflammation (Tilborghs et al., 2017). NF-κB could be activated by almost all infectious agents links with cancer, e.g. human papillomavirus (James et al., 2006), HIV(DeLuca et al., 1996) and Helicobacter pylori (Keates et al., 1997). It was proved that exercise training could prevent tumor-induced TWEAK/NF-κB signaling pathway in skeletal muscle and had a beneficial effect on fiber cross-sectional area and metabolism. This exercise-induced muscle remodeling was related to less malignant mammary lesions in tumor-bearing animals (Padrao et al., 2017).

These findings provide new insights into the potential anti-cancer role of exercise. Since exercise has different parameters, future studies could carry in-depth research of interactions between different mechanisms and try to elucidate the recommended doses and intensities of exercise for cancer, especially in breast, colorectal, prostate cancer and hepatocellular carcinoma.

Compared with other reviews, our study based on Citespace provided a visualized research hotspots and frontiers. However, there are still some limitations in this study. Due to the limitation of Citespace software, we only searched SCI-Expanded and analyzed studies published in English. Therefore, the data may not be comprehensive enough. Articles published in other languages and databases need further research.

## Conclusion

This study analyzed the research hot spots and frontiers of molecular mechanisms of exercise on cancer via CiteSpace. Based on the results, altered metabolism (aerobic glycolysis, insulin resistance, myokines), oxidative stress, gene expression and apoptosis were hot-research mechanisms of exercise on cancer. Emerging research foci of mechanisms were generally around inflammation, epithelial mesenchymal transition and adipokines. In addition, future studies could carry in-depth research of interactions between different mechanisms and try to elucidate the recommended doses and intensities of exercise for cancer, especially in breast, colorectal, prostate cancer and hepatocellular carcinoma.
